# Supplementary material for: Shockwaves Suppress Adipocyte Differentiation via Decrease in PPARγ
Source: Cells. 2020 Jan 9;9(1):166. doi: 10.3390/cells9010166 (PMC7017360; doi:10.3390/cells9010166)
Supplement: Supplementary file 1 [file cells-09-00166-s001.pdf]

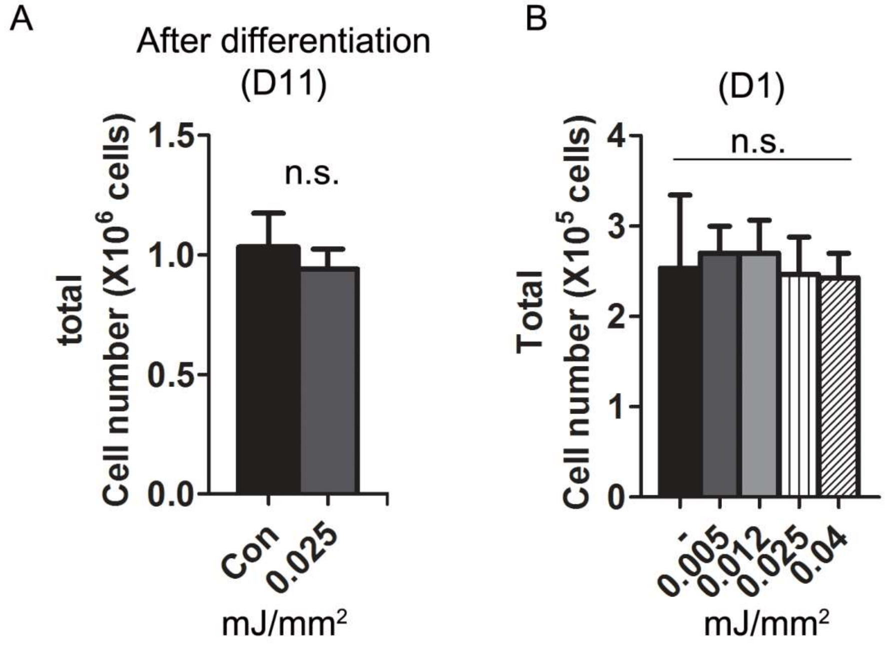

Supplementary Figure 1. There was no difference in total cell number after shockwaves-treatment. (A) After fully differentiation, 5th times shockwave treatment (D11), 0.025 mJ/mm^2 shockwave-treatment resulted in no significant total cell number difference compared with untreated cells. (B) 1st time shockwave (0.005, 0.012, 0.025 and 0.04 mJ/mm^2) treatment (D1) resulted in no significant cell number difference compared with untreated cells. Cells were trypsinized and total cell numbers were counted by Luna™ automated cell counter (Logos Biosystems, Anyang-Si, South Korea).

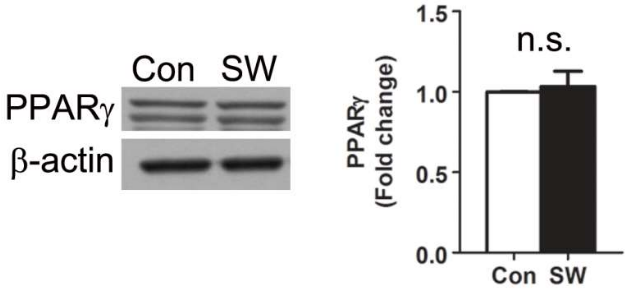

Supplementary Figure 2. Shockwave treatment did not affect the level of PPARγ after fully differentiated 3T3L-1.

3T3L-1 was fully differentiated (D14) and then cells were treated with or without shockwaves. Next day, cells were harvested for immune blot with anti-PPARγ. β-actin was used as internal control. Quantification result of PPARγ (Right graph).
